# Yield Progress in Forage Maize in NW Europe—Breeding Progress or Climate Change Effects?

**DOI:** 10.3389/fpls.2020.01214

**Published:** 2020-08-18

**Authors:** Friedhelm Taube, Iris Vogeler, Christof Kluß, Antje Herrmann, Mario Hasler, Jürgen Rath, Ralf Loges, Carsten S. Malisch

**Affiliations:** ^1^ Kiel University, Grass Forage Science/Organic Agriculture, Christian Albrechts University, Kiel, Germany; ^2^ Grass Based Dairy Systems, Animal Production Systems Group, Wageningen University (WUR), Wageningen, Netherlands; ^3^ Department of Agroecology, Aarhus University, Tjele, Denmark; ^4^ Deutsches Maiskomitee e.V, Bonn, Germany

**Keywords:** plant functional traits, breeding progress, climate change, leaf area index, root biomass, radiation use efficiency

## Abstract

Yield increases in forage maize (Zea mays L.) in NW Europe over time are well documented. The driving causes for these, however, remain unclear as there is little information available regarding the role of plant traits triggering this yield progress. Ten different hybrids from the same maturity group, which have typically been cultivated in Northwest Germany from 1970 to recent and are thus representing breeding progress over four decades, were selected for a 2-year field study in northern Germany. Traits that were investigated included leaf area index, leaf architecture, photosynthesis, radiation use efficiency, root mass, root length density, and turnover. Based on a mixed model analysis with these traits as co-variates, parameters related to leaf characteristics, in particular the number and length of leaves, the radiation use efficiency, and the leaf orientation, were identified as most influential on the yield progress (0.13 tons ha^-1^ year^-1^). In contrast to our hypothesis, root biomass only increased negligibly in newer hybrids compared to older ones, confirming the ‘functional equilibrium’ theory for high input production systems. Due to an abundance of nutrients and water in such high input systems, there is no incentive for breeders to select for carbon partitioning toward the rooting system. Breeding evidence to increase forage quality were also negligible, with no change in cob starch concentration, forage digestibility, nor NDF content and NDF digestibility. The observed increase in yield over the last four decades is due to a combination of increased temperature sums (~240 GDD within 40 years), and a higher radiation interception and radiation use efficiency. This higher radiation interception was driven by an increased leaf area index, with a higher number of leaves (16 instead of 14 leaves within 40 years) and longer leaves of newer compared to older hybrids. Future selection and adaptation of maize hybrids to changing environmental conditions are likely to be the key for high productivity and quality and for the economic viability of maize growing and expansion in Northern Europe.

## Introduction

Introduction Maize (Zea mays L.) is the cereal with the largest global production and is of great economic importance for animal feeding, either as grain or as whole plant forage. Due to the high biomass productivity maize is also increasingly being used for biofuel and biogas production (Herrmann et al., 2014; Pokój et al., 2014; Rath et al., 2015). In the 20-year period between 1991 and 2010, the annual increase in global maize production was 2.2%, achieved through an annual increase in production area of 0.9% and a global average annual yield increase a rate of 1.5%.

In the US and Canada, maize grain yields have increased nearly sixfold during the hybrid era (1939 to present), and according to Lee and Tollenaar (2007), 60% of this increase have been driven by genetic improvements of the hybrids. In Germany, increases in maize grain yields have also been substantial. According to data from FAO (The Food and Agricultural Organization of the United Nations), grain yields have doubled over the 50-year period from 1968 to 2017 (http://www.fao.org/faostat/en/#data/QC). Other factors that have likely contributed to the increases in yield include increased use of fertilizers, better weed control, and improved management practices (Lauer et al., 2001), as well as climate change (Assefa et al., 2012). Increases in yield have also been attributed to higher planting densities, although in NW Europe, these have not changed in the last decades (Dolstra and Miedema, 1986; Finke et al., 1999; LimaGrain, 2011). Several studies on yield trends have been published, but Laidig et al. (2014) pointed out that comparisons of genetic and non-genetic trends should be considered with caution due to interactions of agronomic practices and environmental conditions.

Key genetic improvements, which have driven the increased yield, include (i) more erect leaves in the upper canopy and leaves below the ear horizontally oriented, causing a more even distribution of light within the canopy (Lee and Tollenaar, 2007), as well as a more efficient use of the intercepted light at levels below full sunlight (Pepper et al., 1977); (ii) fast early growth and early flowering; (iii) a longer grain filling period; (iv) increased drought tolerance (Duvick, 2005); and (v) a reduction in the rate of leaf senescence (Frei, 2000). This ‘stay-green’ strategy can result in longer photosynthesis and increased kernel number and weight (Richards, 2000). Other traits that might have contributed to increase the yield are the leaf maximum photosynthesis, and especially a lesser decline of the maximum photosynthesis after silking (Lee and Tollenaar, 2007), as well as increased root growth, enabling improved utilization of often limited resources. For example, studies in China by Ning et al. (2014) and Ning et al. (2015) on six different hybrids released between 1950 and 2008 have found that newer maize hybrids had higher root length at maturity, indicating a lower decrease in root dry matter (DM) after silking. Zhang et al. (2013) also found an initial increase in root mass and root length in hybrids from 1950 to 1980, which was, however, followed by a decrease in the newer hybrids. Wu et al. (2011) found, in a nutrient solution experiment with Chinese maize hybrids released between 1973 and 2009, that root growth only increased under sufficient nitrogen (N) concentrations, but not under N limited conditions. A study in the US by York et al. (2015) on breeding progress of root architecture of maize showed that newer maize cultivars have shallower root angles, fewer nodal roots, and greater distance from nodal roots to lateral branching, potentially enhancing deep resource foraging and N use efficiency. Increases in the belowground biomass are not only important for the supply of water and nutrients but also an important source of stabile soil carbon, with an estimated mean residence time of root-derived carbon at least twice as high as that of shoot-derived carbon (Berti et al., 2016; Komainda et al., 2018).

In Germany and Northwest Europe, maize has commonly been used as silage for cattle feeding, for which, apart from high biomass production, forage quality for ruminant feeding is important. Desirable forage characteristics include high starch concentration for high energy use efficiency in ruminant feeding high organic matter (OM) digestibility and low fiber concentration. Furthermore, for good fermentation and storage, an optimum DM concentration at harvest is required (Lauer et al., 2001). In the last two decades, the maize growing area has rapidly extended toward the north, with a doubling of the growing area, partly due to the promotion of biogas production through subsidies (Claus et al., 2014; Rath et al., 2015).

Intensive silage maize systems in Northwest Europe are associated with high soil OM losses (Komainda et al., 2018b) and high N surpluses (Struck et al., 2019), with a high risk of nitrate leaching losses (Bos et al., 2013). Quantification of carbon inputs through plants residues and roots is important for calculating a carbon balance for these systems, while a high N fertiliser use efficiency (NfUE) is important for protecting our groundwater and limiting further groundwater deterioration (Komainda et al., 2018a; Shepherd et al., 2018). However, information is lacking on the behavior of maize hybrids from different eras on soil organic carbon inputs and NfUE.

Studies using hybrids from different eras and growing these side by side in the same environment for elucidating genetic gains have been made by several researcher (Lauer et al., 2001; Luque et al., 2006; Wang et al., 2011; Badu-Apraku et al., 2015; Li et al., 2015; Ning et al., 2015). Some studies have also been carried out with different planting densities for determining the optimum hybrid-specific density, and to identify if yield increases in newer hybrids are associated with a higher tolerance to density, and this in return to a higher yield stability (Chen K. et al., 2016; Di Matteo et al., 2016). However, no such studies have been done under maritime climate conditions as in NW Europe, where yield and quality increases of maize are mainly determined by the day length and temperature and the resulting length of the vegetation period. Furthermore, none of the above cited studies have used a comprehensive range of functional traits during the crop growth formation for providing insights if and how yield increases have been driven by morphological adaptation.

The objectives of the current study were to determine (i) if and which physiological and morphological traits have driven yield increases and changes in quality in maize hybrids, (ii) if net primary productivity (NPP) increases are evident for both above ground (ANPP) and belowground (BNPP), and (iii) if newer hybrids have a better N use efficiency. We hypothesize that the increases in yield have simultaneously resulted in increases in the root biomass. To address this, 10 different hybrids, which were certified in the period from 1971 to 2012, were grown on field plots over two consecutive years under the same conditions. Furthermore, we quantified the impact of climate change and various traits, including number of plant leaves, leaf area index (LAI), specific leaf area (SLA), leaf architecture (angle and orientation), maximum photosynthesis rate, root growth and turnover rate, root/shoot ratio, and height on silage yield, corn yield, and feeding quality. Measurements were done at flowering and at harvest for silage.

## Materials and Methods

### Study Site Description

The experimental results used are from ongoing field experiments carried out on two fields at the experimental farm ‘Ostenfeld’ (OF), located in the Eastern Upland part of Schleswig-Holstein (54°19’N, 9°48’E). The experiments were carried out over two consecutive years, in 2015 and 2016. To avoid carry over effects and because of the destructive soil sampling for root measurements, two different fields were used in the 2 years. The soil at the side is dominated by a transition between Haplic Luvisol and Cambic Podzol, with loamy sand and sandy loam soils in the two fields.

**Figure 1:**
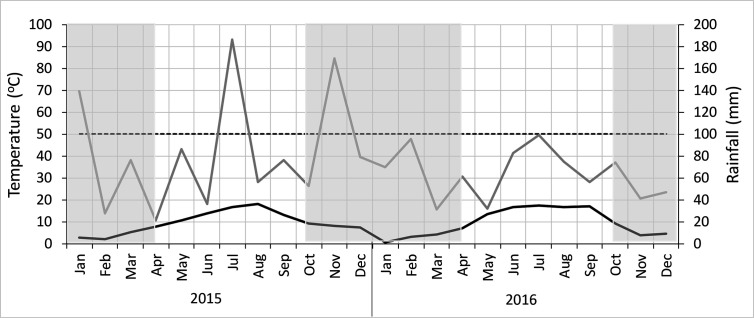
Monthly precipitation (gray line) and monthly average temperature during the experimental period, drawn at the Gaussen scale, following method by (Walter, 1957). Mean monthly rainfall above 100 mm (broken line) indicate a humid period, and drought periods occur when the precipitation undercuts the temperature curve (Richmond and Mueller-Dombois, 1972). Non-growing periods are shaded out.

The region has a temperate oceanic climate with a long-term mean annual temperature of 8.9°C and a long-term mean annual precipitation of 847 mm. Compared with the long-term average, the average temperature was relatively high in both years, with 9.7°C in 2015 and 9.6°C in 2016 (**Figure 1**). Over the growing period (15^th^ April to 15^th^ October), however, 2016 was considerably warmer (14.4°C) than 2015 (13.2°C). The accumulated Growing Degree Days (GDDs) above a base temperature of 6°C were 1,400 for 2015 and 1,650 for 2016. The annual precipitation was considerably higher in 2015 (1,007 mm) and in 2016 slightly lower (766) compared with the long-term average, and during the growing period (April to September), there were 507 (2015) and 457 mm (2016).

**Figure 2:**
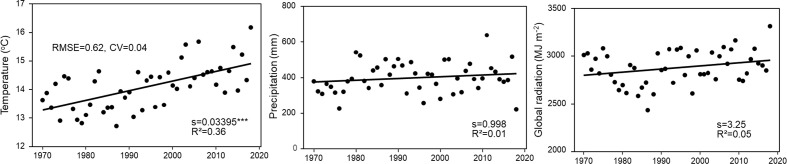
Average annual temperature, precipitation and global radiation from 15^th^ April to 15^th^ October measured at the DWD-Station Kiel-Holtenau. S = slope of the regression. All slopes were tested to be significantly different from zero. Significance level are: ***p < 0.001.

For the modeling of likely climate change impacts on maize yield, data from the DWD-Station (Deutscher Wetterdienst, Germany’s National Meteorological Service) Kiel-Holtenau from 1970 to 2018 were used. While there was a significant increase in the average temperature in the period from 15^th^ April to 15^th^ October, precipitation and global radiation did not change significantly over the 50 year time period (**Figure 2**).

### Maize Hybrids

The selection of the 10 different maize hybrids (**Table 1**) was based on the era in which they were developed (year of release), being representative for the last 40 years (1970 to 2010) in Northwest Germany, and the availability of either the hybrid seeds from the breeders or seeds from parental lines from the gene banks of the breeding companies, which were then used to produce the hybrid seeds. Furthermore, only medium-maturity hybrids with high yield potentials and high feed quality were chosen, apart from Oldham, which is a medium maturity type regarding grain maturity but an early-maturity hybrid regarding silage purposes.

**Table 1:** Details of era representative maize hybrids used in the study.

Hybrid	Year of release	Maturity group	Variety right	Hybrid form	Grain type
Brillant	1971	FAO 230	R.A.G.T. Saaten Deutschland	Double-cross	Intermediate (flint/dent)
Blizzard	1975	S250/K230	Syngenta Agro, Maintal	Three-way	Intermediate (flint/dent)
Tau	1977	S230/K220	Hohenheim/Saaten Union	Three-way	Intermediate (flint/dent)
Mutin	1980	FAO 240	KWS Saat, Einbeck	Three-way	Intermediate (flint/dent)
Beketrio	1990	FAO 230	KWS Saat, Einbeck	Three-way	Intermediate (flint/dent)
Helix	1994	S230/K220	KWS Saat, Einbeck	Single-cross	Intermediate (flint/dent)
Oldham	1999	S220/K230	Syngenta Agro, Maintal	Single-cross	Intermediate (flint/dent)
LG 3232 Lupus	2003	S240/K240	Limagrain,Edemissen	Three-way	flint
Ronaldinio	2006	S240/K240	KWS Saat, Einbeck	Three-way	flint
LG 30224	2012	S 230	Limagrain,Edemissen	Single-cross	flint

The letter of the Reifezahl (maturity index) indicates the use with S = silage maize and K = kernel.

### Experimental Design

The experimental design was a randomized complete block design with three replicated blocks and plot sizes of 9 m × 11 m. Harvest samples were taken from the two central rows of each plot, and for the measurements during the plant development, the other 10 rows were used. To avoid the risk of frost damage to the older, less frost tolerant hybrids, the timing of the sowing (11 May 2015 and 9 May 2016) was slightly later than common practice. The maize was sown at a seed rate of 10 plants m^−2^ and a row distance of 0.75 m. The crops grown prior to the study were winter wheat (*Triticum aestivum*) in 2015 and Sorghum (Sorghum bicolor) in 2016. The fertilization rates were based on common agricultural practices, with a target of 180 kg N ha^-1^, 160 kg P_2_O_5_ ha^-1^, 300 kg K_2_O ha^-1^, and 90 kg ha-1 MgO.

### Measurements

#### Measurements During Crop Development

To identify the physiological and morphological traits that have driven yield increases and changes in quality in maize hybrids, measurements of various plant characteristics were done at mid-anthesis, at a BBCH stage of 65 (**B**iologische Bundesanstalt für Land- und Forstwirtschaft, **B**undessortenamt und **CH**emische Industrie). For further details, see Lancashire et al. (1991). This was on the 14^th^ of August in 2015 and on the 6^th^ of September in 2016. Measurements were done by manually cutting 10 plants per plot to ground level and are referred to as BBCH_65_. Measurements of various aboveground plant characteristics included leaf number (LN), leaf length (LL), leaf angle, LAI, leaf orientation value (LOV), SLA, dry substance (DS), and photosynthesis. The LOV has been developed by Pepper et al. (1977) to describe the plant architecture and the potential for light interception. The LOV takes, apart from the leaf angle at the stem, also the total length of the leaf blade and the length from the leaf collar to the flagging point (Lf) into account.

Photosynthesis rate (PSR) was measured on three plants in per plot and in three levels within the canopy (at, above, and below the cob) *via* a portable Photosynthesis System (LI-6400, LI-COR Inc., USA). The reported results are the averages across these levels.

Apart from these measurements at mid-anthesis and maturity, the LAI and net primary production (ANPP), which are required for the calculation of the radiation use efficiency (see below), were measured during the crop development. These measurements were done on five plants per plot at approximately monthly intervals, in 2015 from BBCH 14 to 75 and in 2016 from BBCH 33 to 71.

Belowground characteristics, which were measured included root length density (RLD) and specific root length (SRL) at BBCH_65 and root growth throughout the season in each of plot of the three blocks. RLD was measured by taking soil cores (h =15 cm, d = 8 cm) to a soil depth of 30 cm, with four replicates per plot. The RLD was then determined using a modified approach of the Newman line-intersect method (Tennant, 1975). After the determination of the RLD, the roots were oven dried at 58°C until constant weight. Prior to analysis, all root samples were milled in a ball-mill (model MM-2; Retsch GmbH, Haan, Germany). Analysis for contents of C- and N followed the Dumas combustion method (Vario Max CN, Elementar, Hanau, Germany). Belowground biomass (root) growth was determined with the ingrowth core method (Steingrobe et al., 2000; Chen S. et al., 2016), with four cores per plot. For this, mesh bags (synthetic fiber net, mesh size of 1 mm, diameter of 4 cm, and length of 60 cm) were filled with pre-sieved (≤1 mm) and root-free soil from the same field and placed into cores, which were installed into the soil at an angle of 45° relative to the soil surface to a vertical depth of 30 cm. Further details are provided in Loges et al. (2018). The four cores were spaced evenly between the plant base and the center of the interrow. The cores were sampled in intervals of four weeks from May to October. During winter, the ingrowth cores were installed in November and remained in the soil until the end of March. After the cores were sampled, the roots were washed over a 0.63 mm sieve and manually separated from other soil constituents (Smucker et al., 1982). Cumulative root growth in the bags over the entire growing period to BBCH85) provided total belowground net primary production (BBCH85_BNPP).

#### Measurements at Maturity

At silage maturity, BBCH stage of 85, which occurred on the 26^th^ of October in 2015 and on the 26^th^ of September in 2016, various plants measurements were done to determine the partitioning of the DM into the various plant parts. These measurements were made on 10 plants per plot, which were manually cut to ground level. To ensure similar crop development, the target DM content for the harvest was set to 300–350 g kg^−1^, the range where the maturity for producing silage is reached (Mikkelsen et al., 2008). These measurements from manual cuts are referred to as BBCH85_M_. The manually harvested plants were separated into vegetative parts (BBCH85_M_vegetative) and cobs (BBCH85_M_cobs).

After this last manual cut, the plots were harvested using a Haldrup harvester to determine plot yields and and forage quality. The stubble height after harvest was 200 mm. The yield from the Haltrup harvest is referred to as BBCH85_H_DMY. Various forage quality measurements, including DM content (g/kg), neutral detergent (NDF) fiber concentration, metabolisable energy (ME), sugar and starch, and C and N concentrations, were determined from these yields and are referred to as BBCH85_H_.

Immediately after harvest, the total fresh matter yield was determined, and the DM content was obtained by oven drying the samples at 60°C for 48 h. For further analysis (forage quality parameters), the materials were milled to pass a 1 mm sieve (Cyclotec mill, Tecator, Foss, Hillerød, Denmark). The C and N contents (g kg^−1^ DM) of samples were determined by the DUMAS combustion method (Vario Max CN, Elementar, Hanau, Germany) and starch (g kg DM^−1^) polarimetrically. Metabolizable energy, N concentrations, and starch were estimated *via* near-infrared reflectance spectroscopy following Herrmann et al. (2014) with a NIR-System 5000 scanning monocrometer (FOSS, Silver Spring, USA). Contents of metabolizable energy (ME) were estimated according to GfE [Gesellschaft für Ernährungsphysiologie] (2008) and Weißbach et al. (1996).The content of NDF (g/kg DM) was determined using the semi-automatic ANKOM 220 Fiber Analyzer (ANKOM Technology, Macedon, USA).

### Data Analysis

The aboveground net primary production (ANPP) was calculated form the sum of DMY and the stubble yield. The net primary production (NPP) was calculated from the sum of ANPP and BNPP, and the ratio (f_BNPP_) between the rootmass and the total NPP as fBNPP = BNPP/NPP.

Nitrogen fertiliser use efficiency (NfUE), N uptake efficiency (N_upt_E), and N yield use efficiency (NYUE) were calculated as:

$$NfUE\;(kgDM/kgN_{f})=\frac{\text{BBCH}85\_H\_DMY}{N_{f}}$$

$$N_{upt}E\;(kgN/kgN_{f})=\frac{\text{BBCH}85\_H\_N_{uptake}}{N_{f}}$$

$$NYUE\;(kgDM/kgN_{uptake})=\frac{\text{BBCH}85\_H\_DMY}{N_{uptake}}$$

where N_f_ is the fertilizer rate, which equaled 180 kg N/ha, and N_uptake_ is the N uptake by the plants measured until BBCH stage of 85 (kg N/ha).

The radiation use efficiency (RUE; g MJ^–1^) was calculated from the measured leaf area development and the following equations:

$$RUE=\sum_{j=1}^{n}\frac{NPP_{j}}{PARi_{j}}$$

where *PAR_ij_* is the intercepted photosynthetic active radiation over the *j*th measurement period (MJ m^-2^), which was estimated using (Varlet-Grancher et al., 1989; Schwerz et al., 2019):

$$PARi_{j}=\sum_{j=1}^{n}0.95\;(1-e^{-a\;LAI_{j}})PAR0_{j}$$

where *PAR*_0,j_ is the incident photosynthetically active radiation [assumed to be 0.5 of the incident solar radiation (Sinclair and Muchow, 1999)], LAI*j* the *j*th observation, and *a* is a crop specific value, which for maize is taken as 0.65 as used in the CERES-Maize model (Jones and Kiniry, 1986).

### Effect of Historic Climate on Maize Yield

While in this study hybrids from the various eras were grown side by side under the same climatic conditions, we investigated the impact on possible past climate change on maize yields based on the MaisProg simulation model (Herrmann et al., 2005; Kruse et al., 2008). For this, two hybrids, Oldham and Ronaldino, were used, and climatic conditions were measured at the DWD-Station Kiel-Holtenau from 1970 to 2018. In the model, growth is calculated based on weather data (daily data of average air temperature, precipitation, potential evapotranspiration, and global radiation) and on plant and soil characteristics. Hybrid-specific growth parameters are the initial biomass, which was set to 0.435 for Oldham and 0.220 for Ronaldino, the young crop’s relative growth rate set to 0.218 for Oldham and 0.240 for Ronaldino, and the shape parameter of the age function set to 2.098 for Oldham and 2.050 for Ronaldino. These parameters were based on calibration datasets described by Herrmann et al. (2005) and Kruse et al. (2008).

### Statistical Analysis

The statistical software R (R Core Team, 2020) was used to evaluate the data. The data evaluation started with the definition of a mixed model for an initial regression analysis (Pinheiro and Bates, 2000; Venables and Ripley, 2002; Pinheiro et al., 2020). This model only included release year as a fixed, quantitative factor. Each release year corresponds to one hybrid. The model is given by:

$$y_{i,j,k}=\mu +\beta \;x_{i}+a_{k}+b_{j,k}+g_{i}+{\left(ga\right)}_{i,k}+e_{i,j,k}$$

where *μ* is the intercept, *β* is the slope, *x*_i_ is the *i*-th release year, *a*_k_ is the random effect of the *k*-th experimental year, *b*_j,k_ is the random effect if the *j*-th block nested in the *k*-th experimental year, *g*_i_ is the random effect of the *i*-th hybrid, (ga)_i,k_ is the random interaction of the *i*-th hybrid and the *k*-th year, and *e*_i,j,k_ is the residual error associated with *y*_i,j,k_.

Based on a graphical residual analysis, the residuals were assumed to be normally distributed and to be homoscedastic. A Pseudo R^2^ was calculated for the mixed model (Nakagawa and Schielzeth, 2013). In a next step, this model was extended to also include a set of co-variates within a multiple regression analysis. The specific co-variates were based on a correlation analysis, the Akaike information criterion value (AIC), and a VIF analysis (Fox and Monette, 1992).

## Results

### Net Primary Production—Above and Belowground

**Figure 3:**
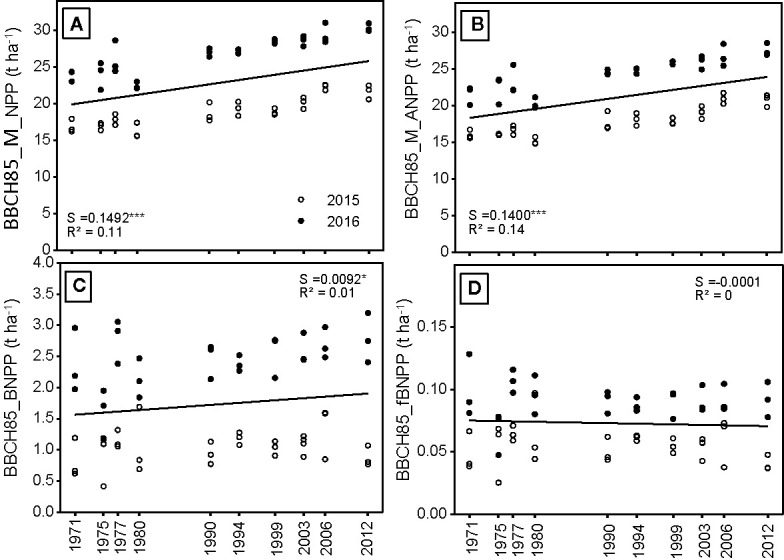
**(A)** Netto Primary Production (H_NPP) at harvest **(B)** aboveground NPP (H_ANPP), **(C)** belowground NPP(H_BNPP), and **(D)** fraction of belowground NPP to total NPP (H_fBNPP) measured in three blocks from 10 different hybrids, which were released in the period between 1971 and 2012 and either grown in 2015 or 2016; S = slope of the regression. The regression indicates differences among hybrids across the year of registration. All slopes were tested to be significantly different from zero. Significance levels are: ***p < 0.001 and *p < 0.05.

The net primary production (BBCH85_M_NPP) of the various maize hybrids were higher when the various hybrids were grown in 2016 compared to 2015 (**Figure 3**). This is due to the better weather conditions in 2016, with a 1.8°C higher average temperature and a more evenly distributed rainfall over the growing period (**Figure 1**). The linear mixed model analysis against release year of the hybrids shows that BBCH85_M_NPP increased significantly with year (**Figure 3** and **Table 2**). Averaged across the 2 years, BBCH85_M_NPP ranged from 19.3 t ha^-1^ to 26 t ha^-1^, with a significant positive trend between 1972 and 2012 and an average increase of about 150 kg ha^-1^ yr^-1^. Almost the entire increase in BBCH85_M_NPP with hybrid release year was due to an increase in the BBCH85_M_ANPP, which increased (averaged over the 2 years) by 140 kg ha^-1^ yr^-1^. Similar to the differences in BBCH85_M_ANPP, root growth (BBCH85_BNPP) was also higher in 2016 compared with 2015. In contrast to our hypothesis, the increase in BBCH85_M_ANPP was not complemented by a simultaneously increase in BBCH85_BNPP (**Figure 3**).

**Table 2:** Mean values (with three replicates and over 2 years) of agronomic and morphological/physiological parameters for the various maize hybrids tested measured at BBCH of 65 or 85 and sorted by YOR.

Factor	1971	1975	1977	1980	1990	1994	1999	2003	2006	2012	slope	R^2^
**Amounts of above ground material**												
BBCH85_M_ NPP (t/ha)	20.38 (1.6)	20.45 (1.66)	21.95 (1.94)	19.3 (1.42)	22.84 (1.89)	23.25 (1.78)	23.65 (2.15)	23.78 (1.71)	25.87 (1.64)	25.99 (1.97)	0.1492***	0.11
BBCH85_M_ ANPP (t/ha)	18.78 (1.28)	19.2 (1.5)	19.98 (1.57)	17.7 (1.17)	21.14 (1.56)	21.47 (1.52)	21.86 (1.81)	22.04 (1.42)	23.85 (1.37)	24.16 (1.55)	0.1400***	0.14
BBCH85_H_DMY (t/ha)	17.77 (1.18)	18.23 (1.43)	19.02 (1.5)	16.63 (1.09)	19.88 (1.41)	20.12 (1.45)	20.81 (1.74)	20.83 (1.33)	22.71 (1.27)	22.89 (1.48)	0.1334***	0.14
BBCH85_M_vegetative_ DMY (t/ha)	4.91 (0.64)	5.77 (0.84)	7.37 (1.09)	5.47 (0.48)	8.29 (1.37)	8.71 (0.76)	8.2 (0.69)	9.35 (0.53)	8.48 (0.95)	10.13 (0.62)	0.1099***	0.34
BBCH85_M_Cob_DM (t/ha)	12.86 (1.54)	12.47 (2.06)	11.65 (1.5)	11.16 (0.72)	11.58 (1.36)	11.41 (0.76)	12.61 (1.46)	11.48 (1.2)	14.23 (1.34)	12.76 (0.9)	0.0235	0.01
BBCH85_M_Stems (g/m^2^)	515.51 (31.72)	543.89 (33.77)	558.27 (33.86)	550.38 (41.53)	650.24 (47.7)	660.42 (21.18)	623.61 (41.12)	666.7 (37.89)	725.18 (33.83)	709.67 (30.18)	4.8130***	0.37
BBCH85_M_Leaf (g/m2)	221.34 (16.29)	207.51 (21.14)	259.26 (20.47)	221.09 (13.38)	253.92 (12.26)	288.72 (18.18)	263.93 (26.31)	304.42 (16.64)	321.56 (11.77)	316.89 (16.47)	2.6340***	0.39
BBCH85_M_Stubble (t/ha)	1.01 (0.11)	0.97 (0.08)	0.96 (0.08)	1.07 (0.08)	1.26 (0.15)	1.35 (0.08)	1.05 (0.09)	1.21 (0.1)	1.14 (0.1)	1.27 (0.08)	0.0066***	0.11
BBCH85_M_height (cm)	262.33 (15.55)	260.67 (15.75)	277.5 (15.64)	249.83 (17.05)	282.5 (18.73)	295.67 (14.83)	282.33 (17.22)	261.71 (11.82)	291 (17.11)	284.83 (15.72)	0.6352***	0.03
**Forage quality**												
BBCH85_H_DM_N (%)	1.31 (0.01)	1.29 (0.01)	1.34 (0.02)	1.33 (0.03)	1.31 (0.03)	1.3 (0.03)	1.23 (0.02)	1.24 (0.02)	1.29 (0.03)	1.22 (0.03)	-0.0022***	0.19
BBCH85_H_DM_ME (%)	10.55 (0.22)	10.72 (0.14)	10.5 (0.2)	10.56 (0.11)	10.32 (0.19)	10.27 (0.19)	10.75 (0.16)	10.62 (0.14)	10.87 (0.17)	10.75 (0.17)	0.005	0.02
BBCH85_H_DM_NDF (%)	47.39 (2.88)	45.46 (2.35)	47.78 (2.79)	48.14 (1.86)	49.34 (2.28)	49.7 (2.44)	44.38 (2.26)	47.33 (2.17)	44.1 (2.12)	45.49 (2.29)	-0.0541	0.01
BBCH85_H_DM _CP (%)	8.19 (0.07)	8.04 (0.07)	8.35 (0.13)	8.3 (0.16)	8.18 (0.17)	8.13 (0.17)	7.68 (0.13)	7.78 (0.14)	8.08 (0.21)	7.59 (0.16)	-0.0137***	0.19
BBCH85_H_DM_DS (kg/kg)	0.32 (0.01)	0.31 (0.01)	0.32 (0.01)	0.29 (0.01)	0.31 (0.01)	0.3 (0.01)	0.29 (0)	0.29 (0.01)	0.31 (0.01)	0.3 (0.01)	-0.0005**	0.07
BBCH85_M_DM_Starch (%)	27.63 (1.17)	28.14 (1.35)	26.72 (1.03)	24.55 (0.57)	24.34 (1.07)	23.28 (1.85)	31.6 (0.97)	26.96 (1.57)	30.57 (0.97)	26.62 (1.22)	0.039	0.02
BBCH85_M_DM_Sugar (%)	4.59 (1.23)	6 (1.16)	4.25 (1.34)	6.83 (1.2)	4.8 (1.48)	5.3 (1.86)	3.62 (0.91)	5.03 (0.68)	4.5 (1.1)	7.09 (0.94)	0.0031	0.00
**Leaf characteristics**												
BBCH85_M_LeafDM (g/m^2^)	17.13 (0.88)	15.91 (0.69)	18.92 (0.92)	15.49 (1.28)	20.13 (0.89)	16.33 (0.73)	22.79 (2.1)	21.13 (0.58)	19.6 (0.97)	26.7 (1.65)	0.1868**	0.34
BBCH65_LN	14.5 (0.22)	13.5 (0.22)	14.5 (0.22)	13.83 (0.17)	14.67 (0.21)	15.5 (0.22)	14.5 (0.22)	15.43 (0.2)	15.5 (0.22)	15.5 (0.22)	0.0404***	0.32
BBCH65_LAI (m^2^/m^2^)	4.61 (0.22)	4.22 (0.17)	4.88 (0.11)	3.41 (0.25)	5.35 (0.17)	4.54 (0.23)	5.41 (0.3)	5.54 (0.14)	5.52 (0.21)	6.1 (0.34)	0.0425***	0.36
BBCH65_SLA (cm^2^/g)	186.5 (7.35)	197.29 (6.37)	179.2 (6.63)	159.98 (2.86)	182.07 (4.65)	174.55 (1.62)	168.86 (12.7)	170.3 (3)	184.02 (10.81)	149.66 (10.06)	-0.5358**	0.11
BBCH65_Leaf_A (°)^avg^	33.11 (1.21)	32.96 (1.18)	32.13 (1.05)	39.48 (0.76)	34.23 (1.19)	35.05 (1.01)	32.87 (0.94)	28.81 (1.04)	27.41 (0.75)	38.45 (0.59)	-0.0415	0.02
BBCH65_Leaf_L (cm) ^avg^	63.31 (1.11)	61.21 (1.28)	66.66 (1.1)	60.32 (1.82)	67.33 (0.32)	71.54 (0.86)	68.97 (1.79)	67.87 (0.78)	73.51 (1.74)	73.42 (0.77)	0.2680***	0.51
BBCH65_Leaf_LOV ^avg^	35.08 (1.59)	31.76 (1.76)	41.17 (1.1)	29.44 (1.38)	41.48 (2.41)	36.89 (0.96)	42.01 (1.91)	49.52 (1.07)	50.26 (2.1)	40 (1.36)	0.3242*	0.36
BBCH65_Leaf_DM (g/m^2^) ^avg^	19.34 (1.66)	17.39 (1.04)	22.32 (2.1)	19.68 (1.04)	24.73 (1.61)	20.5 (1.38)	24.65 (2.13)	24.89 (1.92)	26.01 (1.95)	31.18 (1.35)	0.2481***	0.32
BBCH65_PSR (mmol m^-2^ s^-1^) ^avg^	37.75 (0.99)	42.06 (2.04)	34.65 (1.69)	36.68 (2.93)	38.17 (3.23)	41.44 (3.53)	34.95 (1.32)	41.52 (1.77)	42.51 (2.73)	40.6 (1.96)	0.0869	0.03
RUE (MJ m^-2^)	2.83 (0.24)	3.04 (0.24)	2.86 (0.27)	2.97 (0.31)	3.43 (0.2)	3.46 (0.1)	3.3 (0.18)	3.43 (0.29)	3.67 (0.06)	3.61 (0.27)	0.0199***	0.14
**Cob characteristics**												
BBCH85_M_Cob N (%)	1.51 (0.04)	1.52 (0.03)	1.51 (0.04)	1.47 (0.03)	1.69 (0.07)	1.69 (0.09)	1.23 (0.02)	1.49 (0.03)	1.44 (0.04)	1.3 (0.03)	-0.0039	0.08
BBCH85_M_Cob NDF (%)	15.89 (1.2)	18.42 (1.46)	18.23 (1.08)	19.05 (1.8)	18.24 (1.58)	15.61 (1.01)	17.86 (1.37)	20.97 (1.93)	18.7 (0.92)	18.07 (0.86)	0.0275	0.01
BBCH85_M_Cob CP (%)	9.44 (0.26)	9.5 (0.16)	9.44 (0.24)	9.2 (0.18)	10.54 (0.47)	10.55 (0.56)	7.68 (0.13)	9.34 (0.17)	9 (0.28)	8.15 (0.19)	-0.0246	0.08
BBCH85_M_Cob Starch (%)	58.65 (0.63)	57.33 (1.39)	56.8 (0.89)	55.28 (1.42)	55.75 (1.29)	57.47 (0.71)	57.52 (0.82)	52.5 (1.94)	55.97 (0.84)	56.7 (0.79)	-0.0417*	0.04
BBCH85_M_Cob Sugar (%)	4.75 (0.26)	4.78 (0.23)	5.28 (0.39)	6.05 (0.23)	4.71 (0.43)	4.99 (0.28)	6.78 (0.6)	7.55 (0.51)	6.22 (0.76)	7.34 (0.34)	0.0566**	0.31
**Root Characteristics**												
BBCH85_BNPP (t/ha)	1.6 (0.38)	1.25 (0.22)	1.97 (0.38)	1.6 (0.29)	1.7 (0.35)	1.78 (0.27)	1.79 (0.35)	1.74 (0.31)	2.02 (0.33)	1.83 (0.44)	0.0092	0.02
BBCH85_fBNPP	0.07 (0.01)	0.06 (0.01)	0.09 (0.01)	0.08 (0.01)	0.07 (0.01)	0.07 (0.01)	0.07 (0.01)	0.07 (0.01)	0.08 (0.01)	0.07 (0.01)	-0.0001	0.00
BBCH65_RLD (cm^3^/cm^3^)	1.66 (0.3)	2.64 (0.51)	1.29 (0.18)	2.61 (0.84)	2.16 (0.67)	1.71 (0.28)	2.21 (0.29)	4.29 (0.51)	1.95 (0.35)	1.53 (0.3)	0.0113	0.01
BBCH65_SRL (m/g)	123.26 (16.65)	168.87 (25.01)	151.69 (16.61)	148.39 (25.63)	158.59 (19.87)	103.88 (3.47)	146.06 (16.05)	161.21 (21.1)	165.97 (16.32)	92.03 (11.49)	-0.3874	0.01

Also provided are the mean values over all hybrids, the slope (increase) with time, and R^2^ is pseudoR^2^. M_ indicates manual harvest, and H_ harvest by Haltrup for silage; ^avg^ = average over the various canopy levels. Root characteristics were measured in the top 30 cm. Numbers in brackets are standard errors. Abbreviations are provided in Table A1.

The slope of the regression indicates differences among hybrids across the year of registration. All slopes were tested to be significantly different from zero. Significance levels are: ***p < 0.001, **p < 0.01 and *p < 0.05.

### Partitioning of Aboveground Net Primary Production

**Figure 4:**
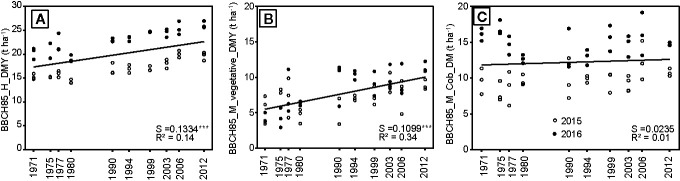
**(A)** Dry matter yield at silage harvest (H_DMY), **(B)** vegetative DMY, and **(C)** cob DM measured in three blocks from 10 different hybrids, which were released in the period between 1971 and 2012 and either grown in 2015 or 2016; S = slope of the regression. The regression indicates differences among hybrids across the year of registration. All slopes were tested to be significantly different from zero. Significance levels are: ***p < 0.001.

The partitioning of the BBCH85_M_ANPP shows that its increase is driven by an increase in the silage yield (**Figure 4**; BBCH85_H_DMY), with an increase of about 130 kg ha^-1^ yr^-1^. This increase was explained to 85% by an increase in the vegetative DM (BBCH85_M_vegetative_DM), with no increase in the cob yield (BBCH85_M_Cob_DM) and only a small increase in the stubble yield of about 6.5 kg ha^-1^ year^-1^ (**Table 2**). Regardless of the hybrid release year, about 30% of the vegetative DM was leaves and the remaining stems.

The biomass DM content at harvest ranged between 260 and 350 g kg^-1^ (**Table 2**). Due to the late development during the early stage of the maize hybrids, the target of 300-350 g kg^-1^ was not reached in 2015, whereas in 2016 all hybrids were on average above 300 g kg^-1^. At flowering the trend in DS with time of release was significantly positive, while at silage harvest the trend was slightly, but significantly (p < 0.05), negative, likely due to the stay-green trait of newer hybrids.

### Plant Traits and Leaf Characteristics

**Figure 5:**
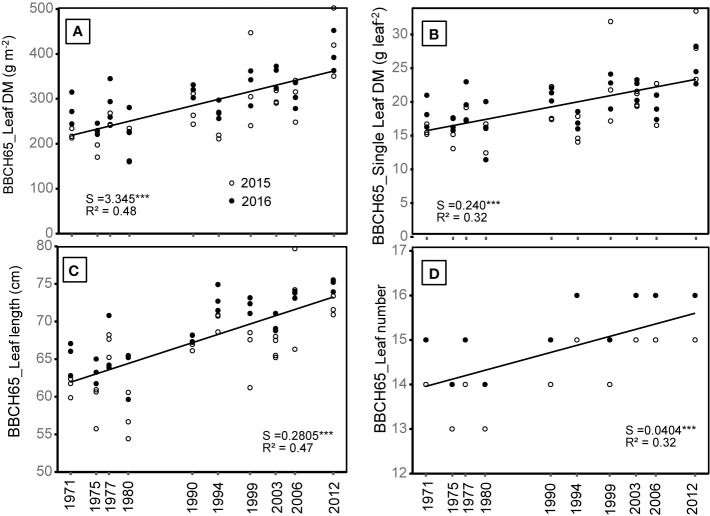
**(A)** Leaf DM (summed over the measurement levels), **(B)** average single leaf DM, **(C)** average Leaf length, and **(D)** average leaf number at flowering from 10 different hybrids, which were released in the period between 1971 and 2012 and either grown in 2015 or 2016. S = slope of the regression. The regression indicates differences among hybrids across the year of registration. All slopes were tested to be significantly different from zero. Significance levels are: ***p < 0.001. Values are averages from three plants per plot with three blocks.

Plant growth and yield are driven by various plant traits, including plant architecture and leaf features. The linear mixed model analysis was used to determine, which plant traits have changed in newer hybrids compared to older ones, and thus potentially contributed to the increase in BBCH85_H_DMY. Leaf characteristics which increased significantly over the years included leaf DM, LL, single leaf DM and LN at BBCH65 (**Figure 5**, **Table 2**).

**Figure 6:**
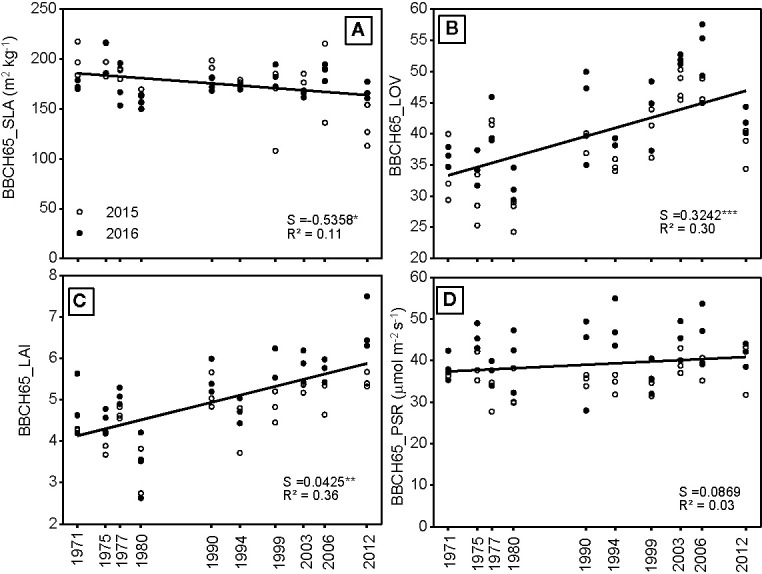
**(A)** Specific leaf area (average over measurement levels), **(B)** LOV, **(C)** LAI, and **(D)** photosynthetic rate at flowering measured on three plants per plot from 10 different hybrids, which were released in the period between 1971 and 2012 and either grown in 2015 or 2016. S = slope of the regression. The regression indicates differences among hybrids across the year of registration. All slopes were tested to be significantly different from zero. Significance levels are: ***p < 0.001 and **p < 0.01, R^2^ = pseudo R^2^. Values are averages from three plants per plot with three blocks.

The SLA showed a small, but significant decrease with release year (**Figure 6**). The increase in LL and the number of leaves resulted in a higher LAI of newer hybrids. Apart from the LAI, the LOV also increased significantly with release year. Both these traits increase the potential for interception of radiation. In contrast, the photosynthetic rate was similar between the various hybrids with no trend.

The radiation use efficiency (RUE) averaged over the measurement period was lower in 2015 compared with 2016. This is likely due to the higher accumulated GDD of 1650 in 2016 compared with a GDD of 1400 in 2015. The fact, that in the first year measurements were done until BBCH 75, whereas in 2016 measurements of LAI and NPP ceased at BBCH71, might also partly explain the lower RUE in 2015. Various studies have shown that RUE decreases during the reproductive phase in many crops, including maize (Subbarao et al., 2005). The average yearly RUE values range from 2.32 to 4.14 g MJ^-1^, and are within reported ranges (Lindquist et al., 2005). For the newer, best performing hybrids RUE values were close to the potential maximum of 4.6 g MJ^-1^ calculated by Loomis and Amthor (1999).

### Forage Quality

**Figure 7:**
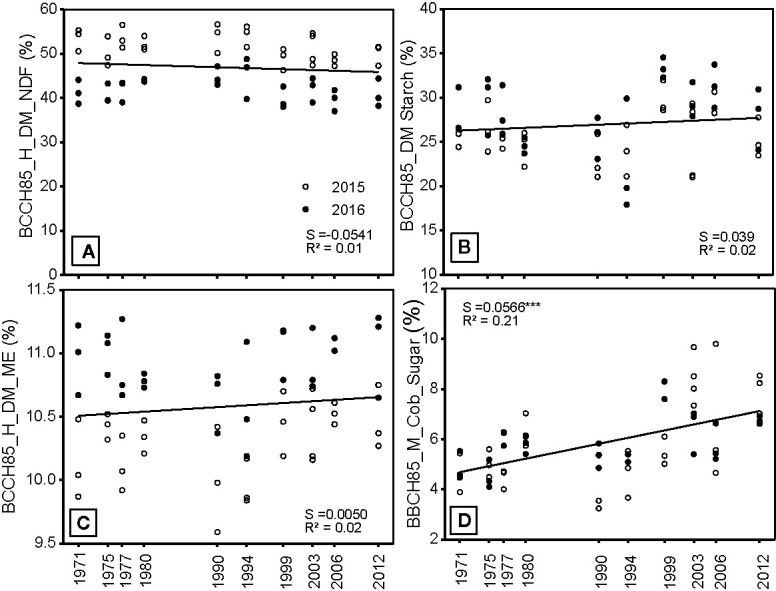
**(A)** Neutral detergent (NDF) fiber concentration **(B)** starch concentration **(C)** metabolizable energy concentration and **(D)** cob sugar concentration of the silage forage measured in three blocks from 10 different hybrids, which were released in the period between 1971 and 2012 and either grown in 2015 or 2016. S = slope of the regression. The regression indicates differences among hybrids across the year of registration. All slopes were tested to be significantly different from zero. Significance levels are: ***p < 0.001.

There was no significant change in forage fiber content and digestability (NDF), forage energy content in terms of ME, nor the starch content with year of release of the hybrids (**Figure 7**). The N- (CP) concentration decreased in newer hybrids, due to a dilution effect (**Table 2**). Similarly, N (RP) concentrations in the cob also decreased in newer hybrids, although this was not significant. This indicates that, in the last four decades, breeding efforts to increase forage quality were negligible apart from the sugar content in the cob, which increased significantly but is more an indicator for sink limitation than of a breeding goal. The feed quality was, however, influenced by different weather conditions in the two experimental years. Whereas the Cob DS (%) was around 60% in 2016, much lower values of between 40 and 50% were obtained in 2015. These low DS were found regardless of the release year, and none of the hybrids achieved the target DS of 58–60%. These results are in line with recent findings, which have shown that environmental conditions have a larger effect on feed quality than the genetic traits of the hybrids (Gruber et al., 2018).

### Nitrogen Use Efficiency

**Figure 8:**
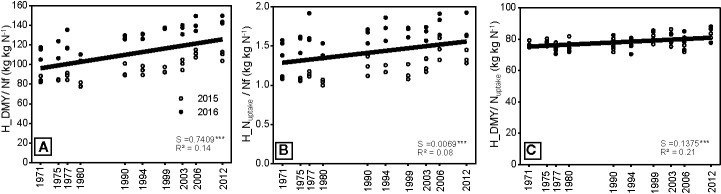
**(A)** Nitrogen fertilizer use efficiency, **(B)** N uptake efficiency, and **(C)** N yield use efficiency measured on three blocks for 10 different hybrids, which were released in the period between 1971 and 2012 and either grown in 2015 or 2016. S = slope of the regression. The regression indicates differences among hybrids across the year of registration. All slopes were tested to be significantly different from zero. Significance levels are: ***p < 0.001.

Nitrogen use efficiency (NfUE), the amount of DM produced per kg N applied, increased with year of release of the hybrid (**Figure 8**), likely due to a higher N demand at higher biomass growth. This is reflected in the higher N uptake efficiency of newer hybrids, as well as a higher N yield use efficiency (NYUE). All hybrids had a slightly higher N uptake then required according to the critical N dilution curve (Lemaire et al., 1996), parameterized for maize growing in Northern Germany by Herrmann and Taube (2004). The ratio between actual N uptake and optimum N uptake ranged between 1.17 and 1.28, with no trend with year of release.

### Effect of Plant Traits on Dry Matter Yield

Correlation analysis indicates that BBCH85_H_DMY is highly correlated with various leaf traits measured at flowering (BBCH65), namely the number of leaves, followed by the LAI, the length of the leaves, and LOV (**Table 3**). Both the leaf angle and the SLA were not significantly correlated with BBCH85_H_DMY.

**Table 3:** Correlation matrix between various plant characteristics at flowering (BBCH65) and the silage DM yield (BBCH85_H_DMY), based on the pearson-method with pairwise-deletion.

BBCH65	DS	LAI	SLA	LL	LOV	LA	Lf	BBCH85_H_DMY
LN	0.57^***^	0.68^***^	-0.19	0.77^***^	0.66^***^	-0.21	0.79^***^	0.78^***^
DS		0.71^***^	-0.67^***^	0.45^***^	0.43^***^	-0.05	0.53^***^	0.40^**^
LAI			-0.06	0.68^***^	0.69^***^	-0.29^*^	0.77^***^	0.69^***^
SLA				-0.13	-0.03	-0.26^*^	-0.16	-0.09
LL					0.60^***^	-0.20	0.84^***^	0.65^***^
LOV						-0.67^***^	0.88^***^	0.59^***^
LA							-0.31^*^	-0.15
Lf								0.72^***^

DS, dry substance; LN, number of leaves; LOV, leaf orientation value; LA, leaf angle; LAI, leaf area index; SLA, specific leaf area; Lf, length from leaf collar to the flagging point. Significant difference at ***p < 0.001, **p < 0.01, and *p < 0.05.

**Figure 9:**
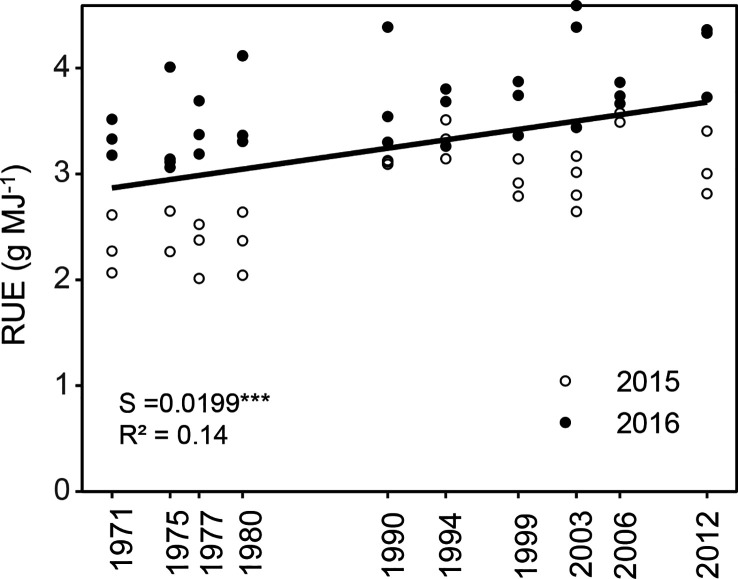
Radiation use efficiency (RUE) from 10 different hybrids calculated from measurements in three blocks, which were released in the period between 1971 and 2012 and either grown in 2015 or 2016. S = slope of the regression. The regression indicates differences among hybrids across the year of registration. All slopes were tested to be significantly different from zero. Significance levels are: ***p < 0.001, R^2^ = pseudo R^2^.

To explore which of these traits identified through the correlation analysis explain the increase in BBCH85_H_DMY with release year, the various traits were included as covariates in the linear mixed model. The explanatory power of the traits was then evaluated based on the AIC values. The LAI and the LAI in combination with the RUE (**Figure 9**), were better at predicting the increase in DMY than the release year of the various hybrids (**Table 4**). None of the other traits (single or in combination) could explain the increase in DMY and the release year, with all traits having higher AIC values.

**Table 4:** Akaike Information Criterion (AIC) values of Release Year (YOR) and various plant characteristics at flowering and the harvested DM yield (H1_DMY), based on the linear mixed model with the plant characteristics as co-variates.

		LAI		LN	LL	Lf	LOV	RUE
AIC	206.39	201.81		213.27	220.48	219.68	219.28	216.53

	LAI+ SLA	LN+ LOV		LN+ Lf	LL+ Lf	LL+ RUE	SLA+ RUE	LN+ LL		LL+ LOV	LAI+ RUE	LN+ RUE	LOV+ Lf	LOV+ RUE	Lf+ RUE
AIC	211.89	216.25		215.38	224.51	221.21	223.5	217.09		222.87	203.2	213.07	224.9	220.44	220.65

	LN+ LL+ LOV	LN+ LL+ Lf	LN+ LL+ RUE		LN+ LOV+ Lf	LN+ LOV+ RUE	LN+ Lf+ RUE	LL+ LOV+ RUE		LAI+ SLA+ RUE
AIC	220.06	220.65	217.29		221.09	216.87	216	224		213.24

LAI, leaf area index; SLA, specific leaf area; LN, number of leaves; LOV, leaf orientation value; LL, leaf length; Lf, length from leaf collar to the flagging point.

## Discussion

### Yield Increases With Hybrid Release Year

Our study, in which hybrids from different eras were grown under the same environment conditions, showed significant increases in BBCH85_H_DMY of newer hybrids compared with older ones. Based on a regression analysis, annual increases in DM with release year equated to 130 kg/ha. Laidig et al. (2014), who compared maize hybrids grown in official variety trials in Germany from 1983 to 2012 reported an annual increase due to due to genetic improvements of 192 kg DM/ha, which is higher compared to the value found in our study. Similarly, Schils et al. (2020) reported a slightly higher annual yield increase based on forage maize variety trials, in which new varieties were compared alongside older ones in the Netherlands from 1991 to 2016. Based on a linear mixed model they estimated a genetic progress of 173 kg DM/ha, and a non-genetic progress of 65 kg DM/ha. The non-genetic progress could be explained by temperature sum during the growing season and the sowing date. However, as pointed out by Fischer (2015) genetic progress estimated from variety trials can be overestimated due to variety aging, with a breakdown of disease resistance.

The observed yield increases in our study were mostly attributable to increases in the above ground vegetative biomass, with no significant changes in the belowground biomass nor the cob yield. The latter suggests that newer hybrids are physiologically less mature, which means that photosynthates are retained in the stems and are not translocated into the cob for the synthesis of starch. Such a ‘storage sink limitation’ can be due to a low assimilate conversion rate from sugars to starch (Engels et al., 2012). Another possibility is that the sink strength of the cob is not high enough for a translocation to take place (Setter and Flannigan, 1986). Future breeding and selection should focus on early starch accumulation to reduce this sink limitation or to increase the sink strength of the cob. Temperature was unlikely a factor for this sink limitation, as critical temperatures of <12°C at which an effective transport of sugar into the cob is limited, only occurred in late September in 2015 and early October 2016.

While our experiments were carried out in Northern Germany, the results should be relevant for a relatively wide latitudinal range, as the cumulative GDD over the two experimental periods were quite different. In 2016, the cumulative GDD was 1697, compared with a GDD of 1,465 GDD in 2015. This difference in in GDD covers according to Spinoni et al. (2015) climatic regions regarding their GDD from Northern France to Southern Denmark.

The focus on breeding or selection on increases in silage yield in Northwest Europe is different to those in warmer and drier climates, where the effort is directed more toward the cob. In a study conducted in three different sites in Wisconsin, Lauer et al. (2001) compared the yield and quality of forage and stover of maize hybrids, which were grown in the Northern Corn Belt during different eras, with the earliest hybrids from 1900 to 1930 to the latest from 1991 to 1998. As in our study, they found significant increases in forage yields. However, these were due to increases in cob yield, with very little changes in stover yield and quality. Similarly, a study from Argentinia showed that yield increases in maize hybrids (from 1965 to 1997) were mainly due to improved kernel number, enhanced post silking biomass production, and enhanced biomass allocation to reproductive sinks (Luque et al., 2006). In contrast, the study by Gonzalez et al. (2018) shows that the maize grain yield increase during the hybrid era in Canada was entirely due an increase in the tolerance to increased plant density, while the individual plant-based yeild potential has remained the same, despite these two attributes not being antagonistic.

### Plant Traits Effects on Yield

The increase in silage BBCH85_H_DMY with release year was best described by LAI and RUE, which both increased with release year. The increase in LAI was due to an increase in the number of leaves and the LL. The increase in RUE at a rate of 0.65% year^-1^ might be due to a more efficient leaf architecture, or to the stay green trait increasing post-anthesis RUE (Tollenaar and Aguilera, 1992). Based on a study in Argentinia, Curin et al. (2020) found very similar increases of 0.7% with hybrids released between 1980 and 2012. This the combined effect of higher radiation interception through the increased LAI and the increase in RUE explain the increase in BBCH85_H_DMY better than release year.

The high influence of LN on the increases in DMY suggests that the breeding processes in the last four decades have been a combination of genetics and environment, especially temperature sum. As maize is very sensitive to low temperatures (<10°C), the longer vegetation period might have endorsed the phenotypic selection of hybrids with higher number of leaves, which require a higher thermal time between sowing and silking (Millner et al., 2005).

Apart from the increase of one to two leaves of newer hybrids, the LL and the LOV also increased significantly. While there was also a tendency in a decrease in the leaf angle, this was not significant as the hybrid from 2012 (LG30224) has a high leaf angle. According to Lee and Tollenaar (2007) the combination of more upright leaves together with a greater LAI has resulted in an 14% increase in the light interception capacity and a >20% increase in yield of modern maize hybrids compared with those released between 1930 and 1960. Steeper leaf angles not only increase the light capture when the sun is at low angles in the sky (morning/afternoon), but also decrease light captures from higher angles (middle of the day and summer), and therefore reduce the susceptibility to photo-inhibition (Werner et al., 2001) and the risk of overheating during the middle of the day. While maize, as a C4 crop, is assumed to have a high temperature optimum, Sinsawat et al. (2004) reported a temporary reduction of leaf photosynthesis of about 50% when leaves of a tropical maize hybrid were exposed to temperatures between 25 and 35°C. Steeper leave angles have also been reported to increase water use efficiency with respect to daily carbon gain (Cowan, 1982; King, 1997). If the observed changes in leaf angle and LAI are sufficient to substantially increase water use, then efficiency requires further investigations. No significant differences in the photosynthetic rate nor a trend with year of release was observed between the various hybrids. Similarly, Richards (2000) stated that, despite intensive selection, the photosynthesis per unit of leaf area in cereals has remained constant or even declined.

### Effects of Release Year on Belowground Biomass

With the worldwide concerns about climate change and global warming, which according to Grace (2004) is to 60% attributable to increasing CO_2_ concentration in the atmosphere, increases in carbon sequestration is important for soil fertility and sustainable crop production. The potential for C sequestration is influenced by climate as well as land use, including crop selection, crop rotations and the use of catch crops, tillage practices, crop residue management, N fertilization and crop yield, and any estimates are highly uncertain. For temperate grasslands annual C sequestrations ranging from 0.1 to 0.5 t/ha have been reported (Grace, 2004; Soussana and Lüscher, 2007). Arable cropping systems often show a decline in SOC (Clivot et al., 2019), particularly when following grassland conversion (Steinmann et al., 2016), and silage maize production systems have been shown to have a negative soil SOM balance (Komainda et al., 2018). Contrary to our hypothesis, root growth was not consistently affected by the release year. As such, newer hybrids are not likely to increase the potential for enhanced carbon sequestration and increases in the stabile soil carbon pool. Apart from inputs from the roots, the SOC balance depends on the difference between losses through erosion and respiration, and inputs through crop residues and organic amendments, and in biofuel production systems also any digestate from the anaerobic digestion, that is returned to the field. As such, the carbon sink of these systems depends on the amounts of digestate and C returned to the field, and by how much this sink is off-set by the loss of C during the anaerobic digestion procedure *via* methane.

The only negligible increase in BNPP, and no other significant changes in root characteristics with release year suggests that root growth, root turnover or greater soil exploration were not thought of as being limiting factors for the breeding or selection of maize hybrids for NW Europe. With the recent focus on climate change mitigation, breeding maize hybrids with denser root systems could simultaneously increase the steady state carbon in the soil and its quality, with better water and nutrient retention, and thus result in more sustainable cropping systems (Kell, 2011).

### Impact of Climate Change on Yield

**Figure 10:**
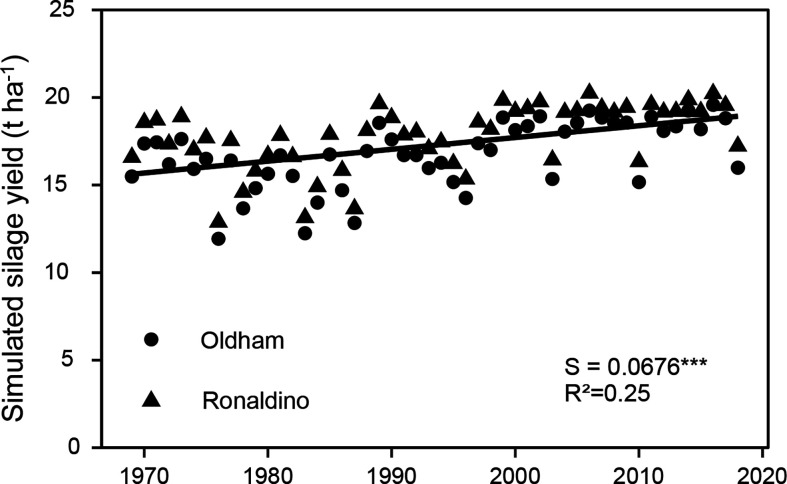
Simulated silage yields for two maize hybrids based on MaisProg simulation model. S = slope of the regression. The regression indicates differences among hybrids across the year of registration. All slopes were tested to be significantly different from zero. Significance levels are: ***p < 0.001.

The differences in BBCH85_H_DMY of the various hybrids between the two study years showed higher variations than those between the various hybrids. This suggests that changes in climatic conditions might be the key driver in the observed increases in maize yield over the last four decades. From 1971 to 2012, average temperatures around Kiel during the growing period have increased by 1.39°C, with no systematic changes in rainfall and global radiation. In line with this, modeling of the historic maize yields of the two hybrids, Oldham and Ronaldino show a significant increase in DMY yield with time, with an average increase of about 68 kg/ha/yr (**Figure 10**). In line with this, Schils et al. (2020) found in maize variety trials in the Netherlands from 1991 to 2016 a non-genetic progress of yields of 65 kg/ha, which was explained by increased temperatures during the growing period, as well as earlier sowing. Several studies have found such a positive effect of climate change on crop production in Northern Europe due to a longer vegetation period with warmer temperatures during the long photoperiod days in high latitude environments in autumn (Rijk et al., 2013).

## Conclusions

The observed increase in silage yield in NW Europe can largely be explained through the increased temperature sum during the vegetation period of maize crops and the resulting earlier maturity in the last four decades. These increased temperatures have a direct effect on the yield, as shown by results from the MaisProg simulation model. According to the model, silage yields increased about 67 kg/ha/year. Apart from this direct effect, the increased temperature also indirectly contributed to the higher yield through the selection or breeding of maize varieties with an increased LAI (higher number of leaves and longer leaves), a higher RUE, and a generally lower leaf angle. Our study showed an annual progress, mainly driven by these plant traits, of about 130 kg DM/ha. The N efficiency of newer hybrids was also higher compared with older ones while forage quality was not affected. In contrast to our hypothesis, root biomass did not increase in newer hybrids compared to older ones.

Selection or breeding of maize hybrids depending on changing environmental condition are likely to be the key for high productivity and quality, as well as for the economic viability of maize growing and expansion in Northern Europe. The recent focus on climate change mitigation and the generally negative SOM balances of silage maize production systems might also shift breeding efforts to maize hybrids with deeper and denser root systems to increase the steady state carbon in the soil.

## Data Availability Statement

Data has been made available at http://dx.doi.org/10.17632/28n4985yyk.1.

## Author Contributions

FT conceived the research idea. JR selected the hybrids and initiated the production from the parental lines. FT, JR, AH, and CM designed the experiments. FT, IV, CK, RL and CM analyzed and discussed the data. AM, RL and CM managed and performed field and lab analysis. MH and CK performed the statistical analysis. FT, IV, and CM wrote the manuscript. All authors contributed to the article and approved the submitted version.

## Funding

The research work was funded by the German Federal Ministry of Food and Agriculture [Bundesministerium für Ernährung und Landwirtschaft (BMEL)] and managed by the Agency for Renewable Resources [Fachagentur Nachwachsende Rohstoffe e.V. (FNR)] (FKZ 22401813; CarboMais: C-Flüsse im Maisanbau).

## Conflict of Interest

The authors declare that the research was conducted in the absence of any commercial or financial relationships that could be construed as a potential conflict of interest.

## Abbreviations

ANPP, aboveground net primary production; BNPP, belowground net primary production; DMY, dry matter yield (t/ha); DM, dry matter; fBNPP, BNPP/NPP; BBCH85_H, measurements done on samples from Haltrup harvester at stage BBCH85; BBCH85_M, measurements done on manually sampled plants at stage BBCH85; BBCH65_, measurements done at BBCH65; DM, dry matter; LA, Leaf angle; LAI, leaf area index; LL, leaf length (cm); LN, leaf number; LW, maximum leaf width; LOV, leaf orientation value; NDF, neutral detergent fiber (%); NPP, net primary production (kg ha^-1^); PSR, Photosynthesis rate (μmol m^-2^ s^-1^); RP, protein (%); RL, root length (cm); RLD, root length density (cm cm^3^); SRL, specific root length (m g^-1^); SLA, specific leaf area (m^2^/kg).

## References

[B1] Assefa Y. Roozeboom K. L. Staggenborg S. A. Du J. (2012). Dryland and irrigated corn yield with climate, management, and hybrid changes from 1939 through 2009. Agron. J. 104, 473–482. 10.2134/agronj2011.0242

[B2] Badu-Apraku B. Fakorede M. A. Oyekunle M. Yallou G. C. Obeng-Antwi K. Haruna A. (2015). Gains in grain yield of early maize cultivars developed during three breeding eras under multiple environments. Crop Sci. 55, 527–539. 10.2135/cropsci2013.11.0783

[B3] Berti A. Morari F. Dal Ferro N. Simonetti G. Polese R. (2016). Organic input quality is more important than its quantity: C turnover coefficients in different cropping systems. Eur. J. Agron. 77, 138–145. 10.1016/j.eja.2016.03.005

[B4] Bos J. F. F. P. Smit A. L. Schröder J. J. (2013). Is agricultural intensification in the Netherlands running up to its limits? NJAS - Wageningen J. Life Sci. 66, 65–73. 10.1016/j.njas.2013.06.001

[B5] Chen K. Camberato J. J. Tuinstra M. R. Kumudini S. V. Tollenaar M. Vyn T. J. (2016). Genetic improvement in density and nitrogen stress tolerance traits over 38 years of commercial maize hybrid release. Field Crops Res. 196, 438–451. 10.1016/j.fcr.2016.07.025

[B6] Chen S. Lin S. Reinsch T. Loges R. Hasler M. Taube F. (2016). Comparison of ingrowth core and sequential soil core methods for estimating belowground net primary production in grass–clover swards. Grass Forage Sci. 71 (3), 515–528. 10.1111/gfs.12214

[B7] Claus S. Taube F. Wienforth B. Svoboda N. Sieling K. Kage H. (2014). Life-cycle assessment of biogas production under the environmental conditions of northern Germany: Greenhouse gas balance. J. Agric. Sci. 152, 172–181. 10.1017/S0021859613000683

[B8] Clivot H. Mouny J. C. Duparque A. Dinh J. L. Denoroy P. Houot S. (2019). Modeling soil organic carbon evolution in long-term arable experiments with AMG model. Environ. Modell. Softw. 118, 99–113. 10.1016/j.envsoft.2019.04.004

[B9] Cowan I. R. (1982). “Regulation of Water Use in Relation to Carbon Gain in Higher Plants,” in Physiological Plant Ecology II. Eds. Lange O. L. Nobel P. S. Osmond C. B. Ziegler H. (Berlin, Heidelberg: Springer Berlin Heidelberg), 589–613.

[B10] Curin F. Severini A. D. González F. G. Otegui M. E. (2020). Water and radiation use efficiencies in maize: Breeding effects on single-cross Argentine hybrids released between 1980 and 2012. Field Crops Res. 246, 107683. 10.1016/j.fcr.2019.107683

[B11] Di Matteo J. A. Ferreyra J. M. Cerrudo A. A. Echarte L. Andrade F. H. (2016). Yield potential and yield stability of Argentine maize hybrids over 45 years of breeding. Field Crops Res. 197, 107–116. 10.1016/j.fcr.2016.07.023

[B12] Dolstra O. Miedema P. (1986). Breeding of silage maize. Proceedings of the 13th Congress of the Maize and Sorghum Section of EUCARPIA (European Association for Research on Plant Breeding) Wageningen, 9–2 September 1985, p. 61–71.

[B13] Duvick D. N. (2005). The Contribution of Breeding to Yield Advances in maize (Zea mays L.). Adv. Agron. 86, 83–145. 10.1016/S0065-2113(05)86002-X

[B14] Engels C. Kirkby E. White P. (2012). Marschners Mineral Nutrition of Higher Plants. 3rd ed. Ed. Marschner P. (San Diego: Academic Press), 85–133.

[B15] Finke C. Möller K. Schlink S. Gerowitt B. Isselstein J. (1999). The environmental impact of maize cultivation in the European Union: Practical options for improvement of the environmental impact-Cast Study Germany. Göttingen, Germany: Research Centre for Agriculture and Environment https://ec.europa.eu/environment/agriculture/pdf/mais_allemange.pdf

[B16] Fischer R. A. (2015). Definitions and determination of crop yield, yield gaps, and of rates of change. Field Crops Res. 182, 9–18. 10.1016/j.fcr.2014.12.006

[B17] Fox J. Monette G. (1992). Generalized collinearity diagnostics. J. Am. Stat. Assoc. 87, 178–183. 10.1080/01621459.1992.10475190

[B18] Frei O. M. (2000). Changes in yield physiology of corn as a result of breeding in northern Europe. Maydica 45, 173–183.

[B19] GfE [Gesellschaft für Ernährungsphysiologie] (1998). Mitteilung des Ausschusses für Bedarfsnormen der Gesellschaft für Ernährungsphysiologie: Formeln zur Schätzung des Gehaltes an Umsetzbarer Energie in Futtermitteln aus Aufwüchsen des Dauergrünlandes und Mais-Ganzpflanzen. Proc. Soc Nutr. Physiol. 7, 141–150.

[B20] Gonzalez V. H. Tollenaar M. Bowman A. Good B. Lee E. A. (2018). Maize yield potential and density tolerance. Crop Sci. 58, 472–485. 10.2135/cropsci2016.06.0547

[B21] Grace J. (2004). Understanding and managing the global carbon cycle. J. Ecol. 92, 189–202. 10.1111/j.0022-0477.2004.00874.x

[B22] Gruber L. Terler G. Knaus W. (2018). Nutrient composition, ruminal degradability and whole tract digestibility of whole crop maize silage from nine current varieties. Arch. Anim. Nutr. 72, 121–137. 10.1080/1745039X.2018.1436665 29458274

[B23] Herrmann A. Taube F. (2004). The range of the critical nitrogen dilution curve for maize (Zea mays L.) can be extended until silage maturity. Agron. J. 96 (4), 1131–1138. 10.2134/agronj2004.1131

[B24] Herrmann A. Kornher A. Taube F. (2005). A new harvest time prognosis tool for forage maize production in Germany. Agr. For. Meteorol. 130, 95–111. 10.1016/j.agrformet.2005.02.005

[B25] Herrmann A. Claus S. Loges R. Kluß C. Taube F. (2014). Can arable forage production be intensified sustainably? A case study from northern Germany. Crop Pasture Sci. 65, 538–549. 10.1071/CP13362

[B26] Jones C. A. Kiniry J. (1986). CERES-Maize. A simulation model of maize growth and development: College Station (Texas: A & M University), 194.

[B27] Kell D. B. (2011). Breeding crop plants with deep roots: Their role in sustainable carbon, nutrient and water sequestration. Ann. Bot. 108, 407–418. 10.1093/aob/mcr175 21813565 PMC3158691

[B28] King D. A. (1997). The functional significance of leaf angle in Eucalyptus. Aust. J. Bot. 45 (4), 619–639. 10.1071/BT96063

[B29] Komainda M. Taube F. Kluß C. Herrmann A. (2018). The effects of maize (Zea mays L.) hybrid and harvest date on above- and belowground biomass dynamics, forage yield and quality - A trade-off for carbon inputs? Eur. J. Agron. 92, 51–62. 10.1016/j.eja.2017.10.003

[B30] Komainda M. Taube F. Kluß C. Herrmann A. (2018a). Effects of catch crops on silage maize (Zea mays L.): yield, nitrogen uptake efficiency and losses. Nutr. Cycling Agroecosys. 110 (1), 51–69. 10.1007/s10705-017-9839-9

[B31] Komainda M. Taube F. Kluß C. Herrmann A. (2018b). The effects of maize (Zea mays L.) hybrid and harvest date on above- and belowground biomass dynamics, forage yield and quality – A trade-off for carbon inputs? Eur. J. Agron. 92, 51–62. 10.1016/j.eja.2017.10.003

[B32] Kruse S. Herrmann A. Kornher A. Taube F. (2008). Evaluation of genotype and environmental variation in fibre content of silage maize using a model-assisted approach. Eur. J. Agron. 28, 210–223. 10.1016/j.eja.2007.07.007

[B33] Laidig F. Piepho H. P. Drobek T. Meyer U. (2014). Genetic and non-genetic long-term trends of 12 different crops in German official variety performance trials and on-farm yield trends. Theoret. Appl. Genet. 127, 2599–2617. 10.1007/s00122-014-2402-z 25307935 PMC4236628

[B34] Lancashire P. D. Bleiholder H. Boom T. V. D. Langelüddeke P. Stauss R. Weber E. (1991). A uniform decimal code for growth stages of crops and weeds. Ann. Appl. Biol. 119 (3), 561–601. 10.1111/j.1744-7348.1991.tb04895.x

[B35] Lauer J. G. Coors J. G. Flannery P. J. (2001). Forage yield and quality of corn cultivars developed in different eras. Crop Sci. 41, 1449–1455. 10.2135/cropsci2001.4151449x

[B36] Lee E. A. Tollenaar M. (2007). Physiological basis of successful breeding strategies for maize grain yield. Crop Sci. 47, 202–215. 10.2135/cropsci2007.04.0010IPBS

[B37] Lemaire G. Charrier X. Hébert Y. (1996). Nitrogen uptake capacities of maize and sorghum crops in different nitrogen and water supply conditions. Agronomie 16 (4), 231–246. 10.1051/agro:19960403

[B38] Li C. F. Tao Z. Q. Liu P. Zhang J. W. Zhuang K. Z. Dong S. T. (2015). Increased grain yield with improved photosynthetic characters in modern maize parental lines. J. Integr. Agric. 14, 1735–1744. 10.1016/S2095-3119(14)60959-X

[B39] LimaGrain U. K. (2011). Maize A Growers Guide. (Limagrain UK Limited).

[B40] Lindquist J. L. Arkebauer T. J. Walters D. T. Cassman K. G. Dobermann A. (2005). Maize radiation use efficiency under optimal growth conditions. Agron. J. 97 (1), 72–78. 10.2134/agronj2005.0072

[B41] Loges R. Bunne I. Reinsch T. Malisch C. Kluß C. Herrmann A. (2018). Forage production in rotational systems generates similar yields compared to maize monocultures but improves soil carbon stocks. Eur. J. Agron. 97, 11–19. 10.1016/j.eja.2018.04.010

[B42] Loomis R. S. Amthor J. S. (1999). Yield potential, plant assimilatory capacity, and metabolic efficiencies. Crop Sci. 39 (6), 1584–1596. 10.2135/cropsci1999.3961584x

[B43] Luque S. F. Cirilo A. G. Otegui M. E. (2006). Genetic gains in grain yield and related physiological attributes in Argentine maize hybrids. Field Crops Res. 95, 383–397. 10.1016/j.fcr.2005.04.007

[B44] Mikkelsen T. S. Hanna J. Zhang X. Ku M. Wernig M. Schorderet P. (2008). Dissecting direct reprogramming through integrative genomic analysis. Nature 454, 49–55. 10.1038/nature07056 18509334 PMC2754827

[B45] Millner J. P. Aver R. V. Hardacre A. K. (2005). The yield and nutritive value of maize hybrids grown for silage. New Z. J. Agric. Res. 48, 101–108. 10.1080/00288233.2005.9513637

[B46] Nakagawa S. Schielzeth H. (2013). A General and Simple Method for Obtaining R^2^ from Generalized Linear Mixed-Effects Models. Methods Ecol. Evol. 4, 133–142. 10.1111/j.2041-210x.2012.00261.x

[B47] Ning P. Li S. Li X. Li C. (2014). New maize hybrids had larger and deeper post-silking root than old ones. Field Crops Res. 166, 66–71. 10.1016/j.fcr.2014.06.009

[B48] Ning P. Li S. White P. J. Li C. (2015). Maize varieties released in different eras have similar root length density distributions in the soil, which are negatively correlated with local concentrations of soil mineral nitrogen. PLoS One 10, e0121892. 10.1371/journal.pone.0121892 PMC4370465 25799291

[B49] Pepper G. E. Pearce R. B. Mock J. J. (1977). Leaf Orientation and Yield of Maize. Crop Sci. 17, 883–886. 10.2135/cropsci1977.0011183X001700060017x

[B50] Pinheiro J. C. Bates D. M. (2000). Mixed-Effects Models in S and S-PLUS (New York: Springer). 10.1007/b98882

[B51] Pinheiro J. Bates D. DebRoy S. Sarkar D. R Core Team (2020). _nlme: Linear and Nonlinear Mixed Effects Models_. R package version 3.1-147.

[B52] Pokój T. Gusiatin Z. M. Bułkowska K. Dubis B. (2014). Production of biogas using maize silage supplemented with residual glycerine from biodiesel manufacturing. Arch. Environ. Prot. 40, 17–29. 10.2478/aep-2014-0035

[B53] R Core Team (2020). R: A language and environment for statistical computing (V., Austria: R Foundation for Statistical Computing). URL https://www.R-project.org/

[B54] Rath J. Heuwinkel H. Taube F. Herrmann A. (2015). Predicting Specific Biogas Yield of Maize-Validation of Different Model Approaches. Bioenergy Res. 8, 832–842. 10.1007/s12155-014-9562-1

[B55] Richards R. (2000). Selected traits to increase crop photosynthesis and yield of grain crops. J. Exp. Bot. 51, 447–458. 10.1093/jexbot/51.suppl_1.447 10938853

[B56] Richmond T. d. A. Mueller-Dombois D (1972). Coastline ecosystems on oahu, hawaii. Plant Ecol. 24, 367–400. 10.1007/BF02675423

[B57] Rijk B. van Ittersum M. Withagen J. (2013). Genetic progress in Dutch crop yields. Field Crops Res. 149, 262–268. 10.1016/j.fcr.2013.05.008

[B58] Schils R. L. M. Van den Berg W. Van der Schoot J. R. Groten J. A. M. Rijk B. Van de Ven G. W. J. (2020). Disentangling genetic and non-genetic components of yield trends of Dutch forage crops in the Netherlands. Field Crops Res. 249, 107755. 10.1016/j.fcr.2020.107755

[B59] Schwerz F. Caron B. O. Elli E. F. Stolzle J. R. Medeiros S. L. P. Sgarbossa J. (2019). Microclimatic conditions in the canopy strata and its relations with the soybean yield. Anais da Acad. Bras. Cienc. 91 (3), e20180066. 10.1590/0001-3765201920180066 31508663

[B60] Setter T. L. Flannigan B. A. (1986). Sugar and Starch Redistribution in Maize in Response to Shade and Ear Temperature Treatment. Crop Sci. 26, 575–579. 10.2135/cropsci1986.0011183X002600030031x

[B61] Shepherd M. Lucci G. Vogeler I. Balvert S. (2018). The effect of drought and nitrogen fertiliser addition on nitrate leaching risk from a pasture soil; an assessment from a field experiment and modelling. J. Sci. Food Agric. 98 (10), 3795–3805. 10.1002/jsfa.8893 29359804

[B62] Sinclair T. R. Muchow R. C. (1999). Radiation Use Efficiency. Adv. Agron. 65, 215–265. 10.1016/S0065-2113(08)60914-1

[B63] Sinsawat V. Leipner J. Stamp P. Fracheboud Y. (2004). Effect of heat stress on the photosynthetic apparatus in maize (Zea mays L.) grown at control or high temperature. Environ. Exp. Bot. 52 (2), 123–129. 10.1016/j.envexpbot.2004.01.010

[B64] Smucker A. J. M. McBurney S. L. Srivastava A. K. (1982). Quantitative Separation of Roots from Compacted Soil Profiles by the Hydropneumatic Elutriation System 1. Agron. J. 74, 500–503. 10.2134/agronj1982.00021962007400030023x

[B65] Soussana J. F. Lüscher A. (2007). Temperate grasslands and global atmospheric change: A review. Grass Forage Sci. 62, 127–134. 10.1111/j.1365-2494.2007.00577.x

[B66] Spinoni J. Vogt J. Barbosa P (2015). European degree-day climatologies and trends for the period 1951-2011. Int. J. Climatol. 35, 25–36. 10.1002/joc.3959

[B67] Steingrobe B. Schmid H. Claassen N. (2000). The use of the ingrowth core method for measuring root production of arable crops - Influence of soil conditions inside the ingrowth core on root growth. J. Plant Nutr. Soil Sci. 163 (6), 617–622. 10.1002/1522-2624(200012)163:6<617::AID-JPLN617>3.0.CO;2-0

[B68] Steinmann T. Welp G. Holbeck B. Amelung W. (2016). Long-term development of organic carbon contents in arable soil of North Rhine-Westphalia, German -2015. Eur. J. Soil Sci. 67, 616–623. 10.1111/ejss.12376

[B69] Struck I. J. Reinsch T. Herrmann A. Kluß C. Loges R. Taube F. (2019). Yield potential and nitrogen dynamics of no-till silage maize (Zea mays L.) under maritime climate conditions. Eur. J. Agron. 107, 30–42. 10.1016/j.eja.2019.04.009

[B70] Subbarao G. V. Ito O. Berry W. (2005). Handbook of Photosynthesis. Ed. Pessarakli M. (New York: Taylor and Francis), 549–576.

[B71] Tennant D. (1975). A Test of a Modified Line Intersect Method of Estimating Root Length. J. Ecol. 63, 995–1001. 10.2307/2258617

[B72] Theuerl S. Herrmann C. Heiermann M. Grundmann P. Landwehr N. Kreidenweis U. (2019). The future agricultural biogas plant in Germany: A vision. Energies 12, 396. 10.3390/en12030396

[B73] Tollenaar M. Aguilera A. (1992). Radiation use efficiency of an old and a new maize hybrid. Agron. J. 84, 536–541. 10.2134/agronj1992.00021962008400030033x

[B74] Varlet-Grancher C. Gosse G. Chartier M. Sinoquet H. Bonhomme R. Allirand J. M. (1989). Mise au point: Rayonnement solaire absorbé ou intercepté par un couvert végétal. Agronomie 9, 419–439. 10.1051/agro:19890501

[B75] Venables W. N. Ripley B. D. (2002). Modern Applied Statistics with S. 4th Edition (New York: Springer). 10.1007/978-0-387-21706-2

[B76] Walter H (1957). Wie kann man den Klimatypus anschaulich darstellen? Umschau die Umschau in Wissenschaft und Technik. 57, 751–753.

[B77] Wang T. Ma X. Li Y. Bai D. Liu C. Liu Z. (2011). Changes in yield and yield components of single-cross maize hybrids released in China between 1964 and 2001. Crop Sci. 51, 512–525. 10.2135/cropsci2010.06.0383

[B78] Weißbach F. Schmidt L. Kuhla S. (1996). Vereinfachtes Verfahren zur Berechnung der NEL aus der umsetzbaren Energie. Proc. Soc Nutr. Physiol. 5, 117.

[B79] Werner C. Ryel R. J. Correia O. Beyschlag W. (2001). Effects of photoinhibition on whole-plant carbon gain assessed with a photosynthesis model. Plant Cell Environ. 24, 27–40. 10.1046/j.1365-3040.2001.00651.x

[B80] Wu Q. Chen F. Chen Y. Yuan L. Zhang F. Mi G. (2011). Root growth in response to nitrogen supply in Chinese maize hybrids released between 1973 and 2009. Sci. China Life Sci. 54, 642–650. 10.1007/s11427-011-4186-6 21748587

[B81] York L. M. Galindo-Castañeda T. Schussler J. R. Lynch J. P. (2015). Evolution of US maize (Zea mays L.) root architectural and anatomical phenes over the past 100 years corresponds to increased tolerance of nitrogen stress. J. Exp. Bot. 66 (8), 2347–2358. 10.1093/jxb/erv074 25795737 PMC4407655

[B82] Zhang F. L. Niu X. K. Zhang Y. M. Xie R. Z. Liu X. Li S. K. (2013). Studies on the Root Characteristics of Maize Varieties of Different Eras. J. Integr. Agric. 12, 426–435. 10.1016/S2095-3119(13)60243-9

